# ROS in diabetic atria regulate SK2 degradation by Atrogin-1 through the NF-κB signaling pathway

**DOI:** 10.1016/j.jbc.2024.105735

**Published:** 2024-02-08

**Authors:** Jian Xu, Dong Zhang, Yibo Ma, Hui Du, Yi Wang, Wenping Luo, Ruxing Wang, Fu Yi

**Affiliations:** 1 Department of Cardiovascular Diseases, Xijing Hospital, Fourth Military Medical University, Xi’an, China; 2 Senior Department of Cardiology, The Sixth Medical Center of PLA General Hospital, Beijing, China; 3 Institute of Cardiovascular and Vascular Disease, Shaanxi University of Traditional Chinese Medicine, Xianyang, China; 4 Department of Cardiology, Wuxi People's Hospital Affiliated to Nanjing Medical University, Wuxi, Jiangsu, China

**Keywords:** diabetes mellitus, atrial fibrillation, NF-κB, Atrogin-1, SK2, ROS

## Abstract

One of the independent risk factors for atrial fibrillation is diabetes mellitus (DM); however, the underlying mechanisms causing atrial fibrillation in DM are unknown. The underlying mechanism of Atrogin-1–mediated SK2 degradation and associated signaling pathways are unclear. The aim of this study was to elucidate the relationship among reactive oxygen species (ROS), the NF-κB signaling pathway, and Atrogin-1 protein expression in the atrial myocardia of DM mice. We found that SK2 expression was downregulated comitant with increased ROS generation and enhanced NF-κB signaling activation in the atrial cardiomyocytes of DM mice. These observations were mimicked by exogenously applicating H_2_O_2_ and by high glucose culture conditions in HL-1 cells. Inhibition of ROS production by diphenyleneiodonium chloride or silencing of NF-κB by siRNA decreased the protein expression of NF-κB and Atrogin-1 and increased that of SK2 in HL-1 cells with high glucose culture. Moreover, chromatin immunoprecipitation assay demonstrated that NF-κB/p65 directly binds to the promoter of the *FBXO32* gene (encoding Atrogin-1), regulating the *FBXO32* transcription. Finally, we evaluated the therapeutic effects of curcumin, known as a NF-κB inhibitor, on Atrogin-1 and SK2 expression in DM mice and confirmed that oral administration of curcumin for 4 weeks significantly suppressed Atrogin-1 expression and protected SK2 expression against hyperglycemia. In summary, the results from this study indicated that the ROS/NF-κB signaling pathway participates in Atrogin-1–mediated SK2 regulation in the atria of streptozotocin-induced DM mice.

The global prevalence of diabetes among individuals aged 20 to 79 years is high and is projected to increase from 10.5% in 2021 to 12.2% in 2045 (1). Diabetes mellitus (DM) increases the risks of cardiovascular disease, including heart failure, dyslipidemia, stroke, hypertension, myocardial infarction, and cardiovascular death. DM is considered an independent risk factor highly associated with atrial fibrillation (AF) (2, 3). However, the mechanisms that modulate atrial electrophysiology in DM are poorly understood.

Small-conductance Ca^2+^-activated K^+^ (SK) channels are widely expressed in the atria (4, 5). There is growing evidence that SK channel expression and function is critical for maintaining normal cardiac rhythm and preventing atrial arrhythmias (6, 7). The ubiquitin‒proteasome system (UPS) is the primary mechanism responsible for the degradation of cellular proteins. The specificity of UPS-mediated protein degradation is determined by ubiquitin E3 ligases (8). Atrogin-1, an ubiquitin E3 ligase, is predominantly expressed in cardiac myocytes. We have previously reported SK2, but not SK1 and SK3, is closely regulated by Atrogin-1 expression with the UPS-dependent manner in the atrial myocardium, contributing to atrial arrhythmias in DM mice (6, 9). However, underlying signaling pathway leading to Atrogin-1 upregulation in DM has not been thoroughly understood.

The reactive oxygen species (ROS) have emerged as critical molecules for the development of diabetic myocardial diseases (10, 11). The transcription factor NF-κB complex, composed of p65 (RelA), RelB, c-Rel, and p50/p105 subunits (NF-κB1) or p52/p100 (NF-κB2), is inactive when bound to its cytoplasmic endogenous inhibitor (IκB) (10). Under different stress signals, including ROS, NF-κB dissociates from IκB, allowing the p50 and p65 translocation to the nucleus to exhibit its transcriptional effect (10, 12). However, the precise mechanism leading to Atrogin-1 upregulation by ROS is unclear. In this study, we tested our hypothesis that ROS/NF–κB/Atrogin-1 axis plays an important role in regulating SK2 expression in atrial cardiomyocytes in DM. Results from this study may help us develop a novel strategy for the treatment of atrial arrythmias in DM.

## Results

### The NF-κB/P65 signaling pathway is activated in the atrial myocardium of DM mice

**Figure 1:**
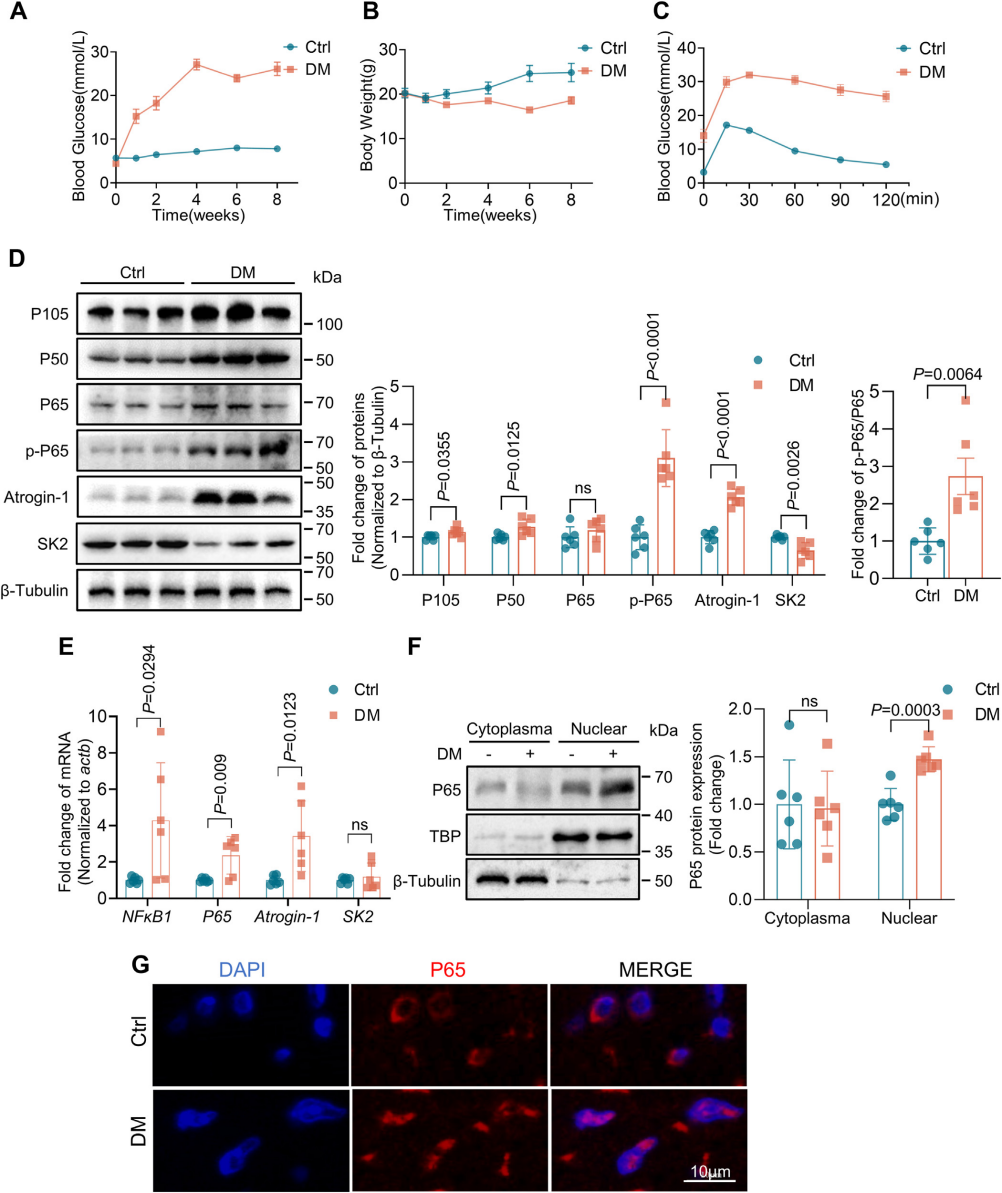
**The NF-κB/P65 signaling pathway is activated in the atrial myocardium of DM mice.***A* and *B*, blood glucose levels and body weights of Ctrl *versus* DM mice at 0 to 8 weeks (n = 10). *C*, glucose tolerance test results for Ctrl *versus* DM mice 2 weeks after i.p. injection of STZ (n = 10). *D*–*G*, C57BL/6J mice 8 weeks after received 125 mg/kg STZ injection (i.p.). *D*, Western blotting analysis of p105, p50, p65, p-p65, Atrogin-1, and SK2 protein expression in Ctrl *versus* DM mice (n = 6). *E*, RT-PCR analysis of NF-κB1, p65, Atrogin-1, and SK2 expression in Ctrl *versus* DM mice (n = 6). *F*, Western blotting analysis of p65 protein nucleoplasmic translocation in Ctrl *versus* DM mice (n = 6). *G*, immunofluorescence analysis of p65 protein nuclear translocation. Data are expressed as the mean ± SD and were assessed by unpaired, two-tailed Student’s *t* test, followed by Bonferroni correction (GraphPad Prism software). DM, diabetes mellitus; SK, small-conductance Ca^2+^-activated K^+^; STZ, streptozotocin.

To explore the signaling pathway that regulates Atrogin-1 expression in the atria of diabetic mice, streptozotocin (STZ)-induced diabetic mice, a well-established type 1 diabetes mouse model characterized by the development of hypoinsulinemia weight loss and elevated blood glucose levels 2 or 3 weeks after STZ injection, were used (Fig. 1, *A*–*C*). Western blotting analysis revealed an increase in the expression of p105, p50, and Atrogin-1 proteins, as well as the phosphorylation of p65 protein (p-p65), accompanied by a marked decrease in SK2 protein expression in the atria of diabetic mice (Fig. 1*D*). To verify whether the changes in protein expression were due to alteration in their mRNA levels, RT-PCR was performed. There was a significant increase in the mRNA levels of NF-κB1, p65, and Atrogin-1, but not in that of SK2 (Fig. 1*E*). Additionally, immunoblotting and immunofluorescence staining experiments showed that DM promoted the translocation of p65-NF-κB from the cytoplasm to the nucleus (Fig. 1, *F* and *G*). These results indicated that enhanced Atrogin-1 expression was contributed to NF-κB signaling activation in diabetic mouse atria.

### Diabetes contributes to increased atrial oxidative stress

**Figure 2:**
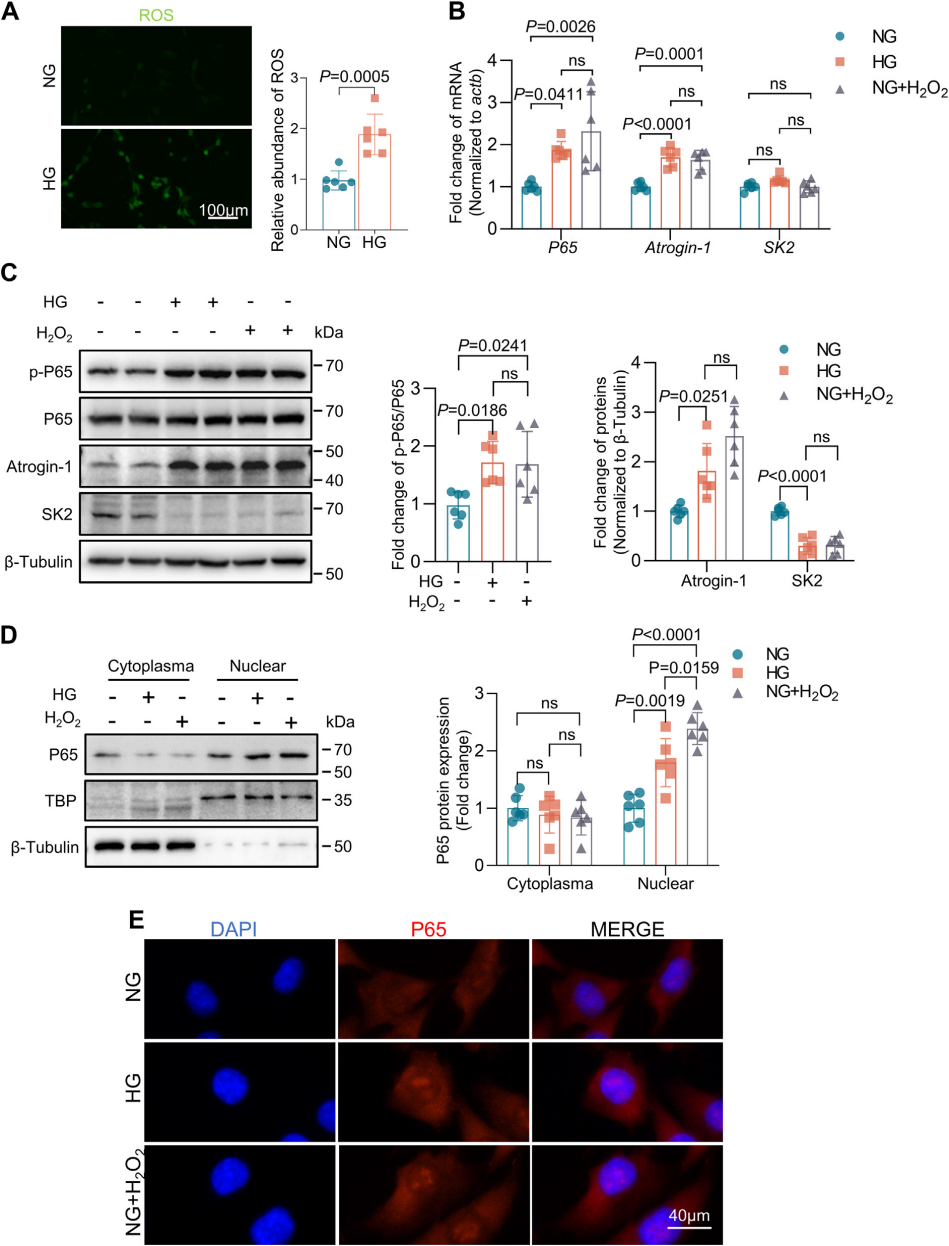
**Diabetes contributes to increased atrial oxidative stress.** Cells were propagated in NG (5 mM) and HG (25 mM) culture media for 14 days; the NG + H_2_O_2_ group was stimulated with 50 μM H_2_O_2_ for 24 h. *A*, HL-1 cells were cultured for 14 days, and intracellular ROS levels were assessed by DCFH-DA (n = 6). *B*, HL-1 cells were propagated in NG or HG medium for 14 days and were stimulated with 50 μM H_2_O_2_ for 24 h before RT-PCR analysis. p65, Atrogin-1, and SK2 expression by RT-PCR after 24 h of H_2_O_2_ stimulation (n = 6). *C*, Western blotting analysis of p-p65 (Ser536), p65, Atrogin-1, and SK2 protein expression (n = 6). *D*, Western blotting analysis of p65 nuclear translocation (n = 6). *E*, immunofluorescence analysis of p65 protein nuclear translocation. Data are expressed as the mean ± SD and were assessed by unpaired, two-tailed Student’s *t* test, followed by Bonferroni correction (GraphPad Prism software). DCFH-DA, dichlorofluorescein diacetate; HG, high glucose; NG, normal glucose; ROS, reactive oxygen species.

Fluorescent probe assay (dichlorofluorescein diacetate) revealed a substantial increase in ROS levels in HL-1 cells cultured with 25 mM glucose (high glucose, HG), compared to those in cells cultured with 5 mM glucose (normal glucose, NG) (Fig. 2*A*). To further identify determine the role of ROS in the activation of NF-κB signaling pathway under HG culture conditions, we measured the mRNA and protein expressions of p65, Atrogin-1, and SK2 in HL-1 cells under the following culture conditions: NG, NG+50 μM H_2_O_2_, and HG 14 days (13). There was a significant increase in the protein levels of p-p65 (Ser536) and Atrogin-1, concomitant with as well as the photoheated protein levels of p65 (Fig. 2*B*), in HG cells, similar to those observed in NG cells with H_2_O_2_, in comparison with NG cells alone (Fig. 2*C*). These results indicate that the NF-κB signaling pathway is activated by HG culture through increased ROS production, causing upregulation of Atrogin-1 protein expression in HL-1 cells.

### Regulation of NF-κB/P65 and Atrogin-1 expression by ROS in HG culture and DM

**Figure 3:**
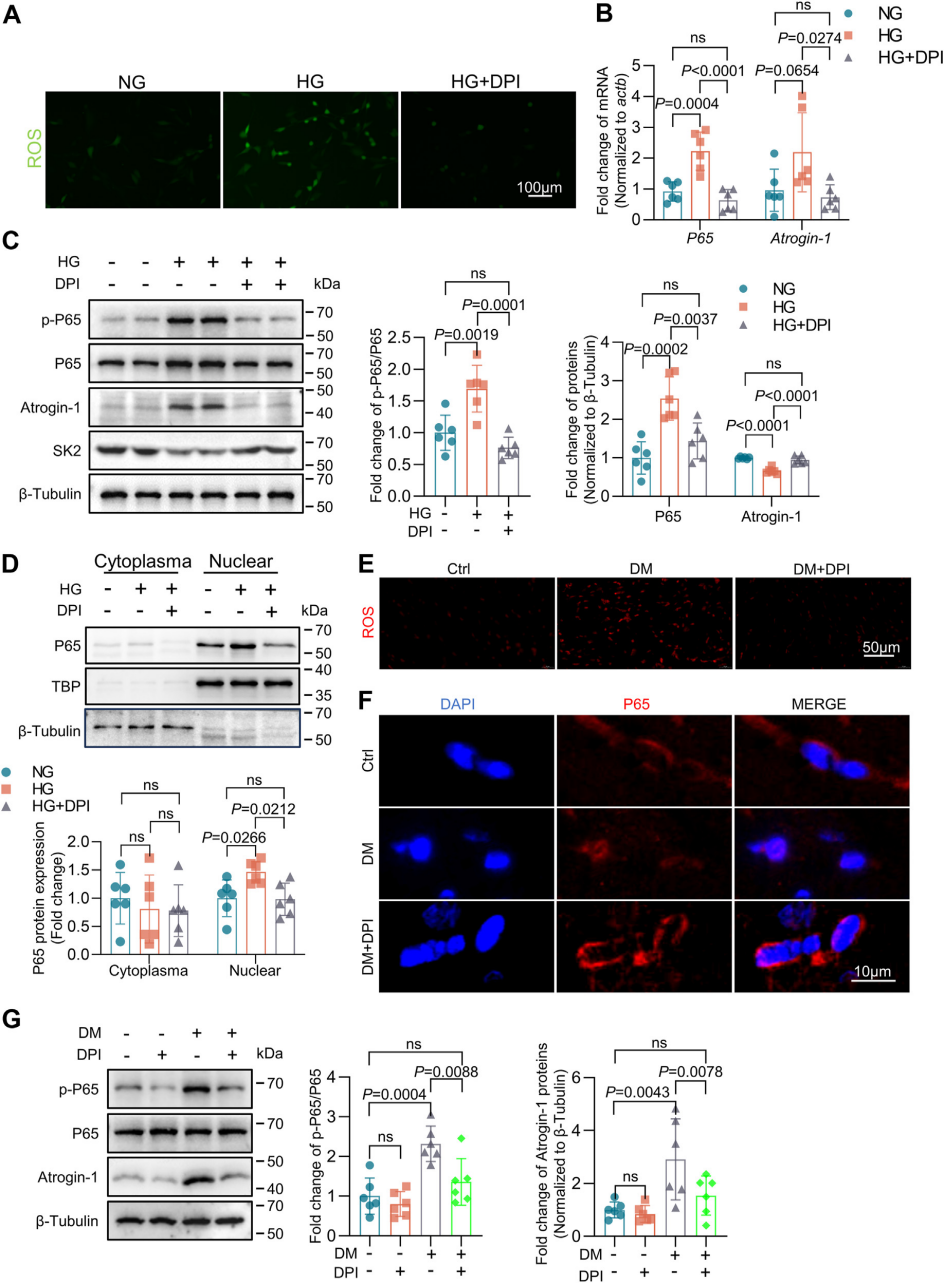
**Regulation of NF-κB/P65 activation by ROS in diabetic atria.***A*–*D*, *in vitro* DPI treatment (10 μM) of HL-1 cells propagated in HG for 24 h. *A*, application of DCFH-DA to assess intracellular ROS content. *B*, RT-PCR analysis of p65 and Atrogin-1 mRNA expression (n = 6). *C*, Western blotting analysis of p-p65 (Ser536), p65, Atrogin-1, and SK2 protein expression (n = 6). *D*, Western blotting analysis of intranuclear p65 expression (n = 6). *E*–*G*, *in vivo* experiments with STZ-induced DM mice, which were administered 0.1 mg/kg DPI, (i.p.) once daily for 2 weeks. DHE fluorescence was applied to assess ROS content in mouse atrial muscle (n = 6). *F*, immunofluorescence to analyze p65 protein nucleoplasmic translocation (n = 6). *G*, Western blotting analysis of the effect of DPI on p-p65 (Ser536), p65, and Atrogin-1 protein expression in mouse atrial muscle (n = 6). Data are expressed as the mean ± SD and were assessed by unpaired, two-tailed Student’s *t* test, followed by Bonferroni correction (GraphPad Prism software). DCFH-DA, dichlorofluorescein diacetate; DHE, dihydroethidium; DM, diabetes mellitus; DPI, diphenyleneiodonium chloride; HG, high glucose; ROS, reactive oxygen species; STZ, streptozotocin.

We assessed whether application of diphenyleneiodonium chloride (DPI) (10 μM, 24 h) prevents p65 phosphorylation and inhibits the increase in Atrogin-1 expression in HL-1 cells in HG culture indeed, application of DPI suppresses ROS generation to the levels of HG-cultured cell, and it reduced the p65 phosphorylation and nuclear translocation, resulting in downregulation of the mRNA and protein levels of Atrogin-1 (Fig. 3, *A*–*D*). These results were confirmed in DM mice by received DPI injections (0.1 mg/kg, i.p.) for 2 weeks, which suppressed atrial ROS generation (Fig. 3*E*), decreased the p65 phosphorylation and nuclear translocation, and downregulated Atrogin-1 expression in the atria (Fig. 3, *F* and *G*). Thus, activation of NF-κB signaling pathway and upregulation of Atrogin-1 protein levels are associated with increased oxidative stress in the atria of DM mice.

### Regulation of SK2 expression by NF-κB/Atrogin-1 axis

**Figure 4:**
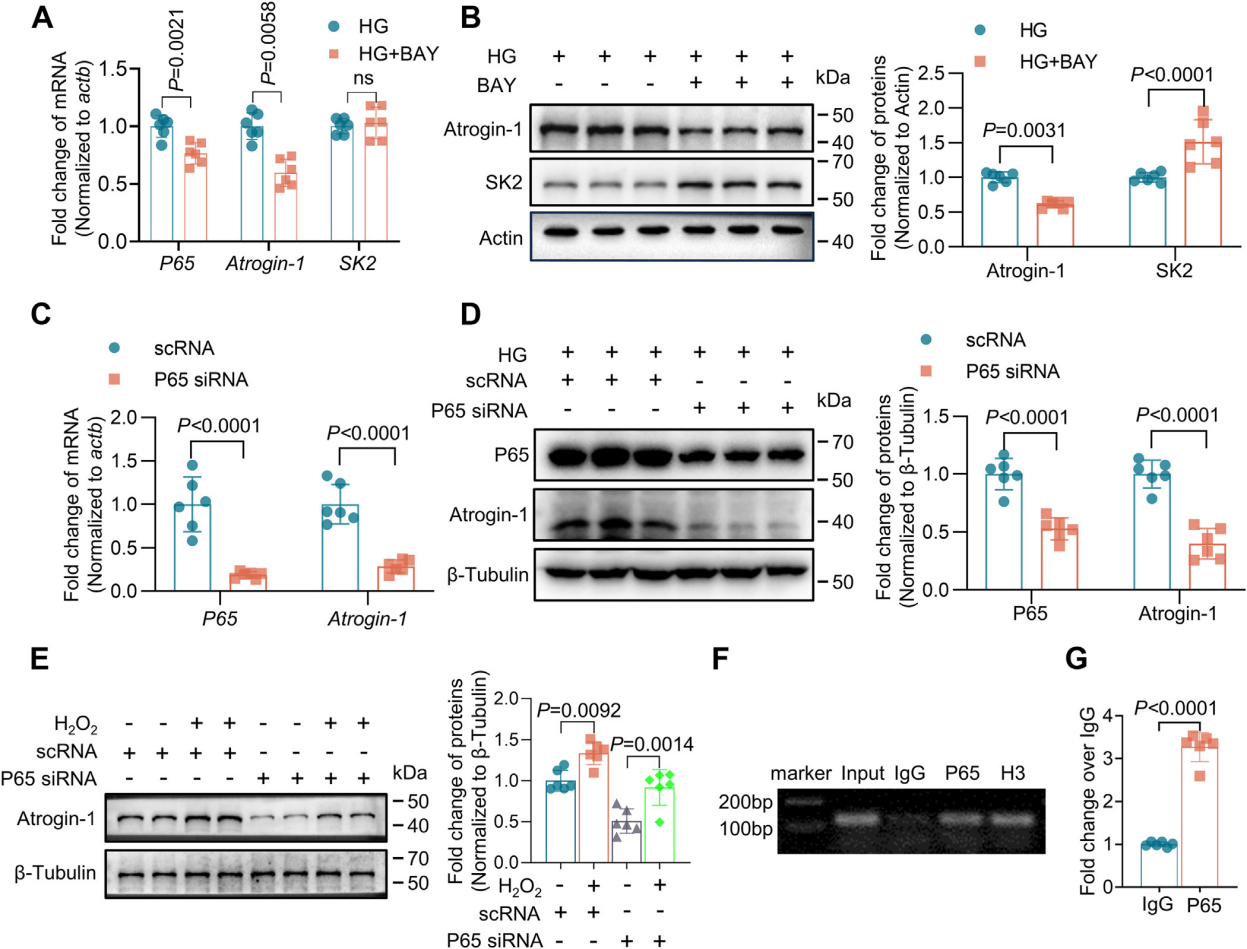
**NF-κB/P65 regulates Atrogin-1 protein changes.***A*, HG-cultured HL-1 cells were treated with BAY11-7082 (5 μM) for 12 h. RT-PCR analysis of p65, Atrogin-1, and SK2 mRNA expression (n = 6). *B*, Western blotting analysis of BAY11-7082 on Atrogin-1 and SK2 protein expression in HG-cultured HL-1 cells (n = 6). *C* and *D*, application of siRNA for the knockdown of the p65 for 48 h. *C*, RT-PCR analysis of p65 and Atrogin-1 mRNA expression (n = 6). *D*, Western blotting analysis of p65 and Atrogin-1 protein expression after p65 gene knockdown (n = 6). *E*, NG-cultured HL-1 cells were treated with p65 siRNA for 48 h and then with H_2_O_2_ (50 μM) for 24 h. Western blotting analysis of Atrogin-1 protein expression (n = 6). *F* and *G*, HG-cultured HL-1 cells were subjected to ChIP using a p65 antibody. *F*, the HG-cultured HL-1 cells were incubated with IgG (negative control), anti-p65 (target gene) or anti-H3 (positive control) antibody, PCR amplification, followed by agarose gel electrophoresis analysis of Atrogin-1 promoter sequence-enriched fragments, (n = 6). *G*, the HG-cultured HL-1 cells were incubated with IgG or anti-p65 antibody, ChIP-qPCR was employed to assess the p65 occupancy on Atrogin-1 promoters, (n = 6). Data are expressed as the mean ± SD and were assessed by unpaired, two-tailed Student’s *t* test or ANOVA, followed by Bonferroni correction (GraphPad Prism software). ChIP, chromatin immunoprecipitation; HG, high glucose; NG, normal glucose; SK, small-conductance Ca^2+^-activated K^+^.

**Supplementary Figure 1:**
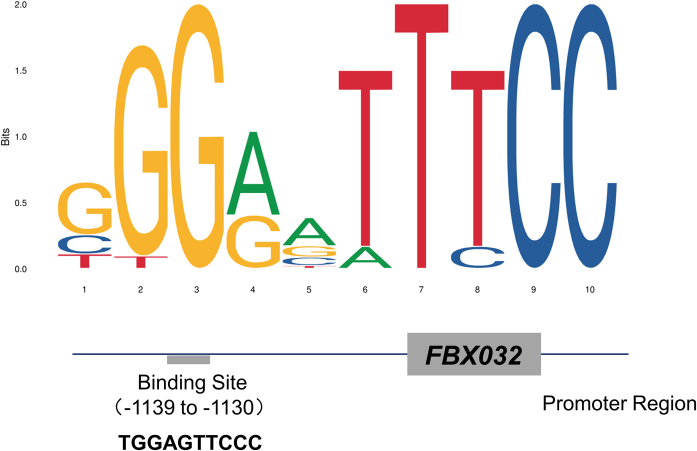
Consensus binding motif of p65 and predicted binding sites at the Atrogin-1 locus.

The role of NF-κB/p65 in Atrogin-1 expression was further assessed using NF-κB inhibitor MAY 11-7082. HL-1 cells treated with 5 μM BAY 11-7082 in HG reduced the mRNA and protein expression of Atrogin-1 (Fig. 4, *A* and *B*). Although no significant effect on SK2 mRNA expression was observed, the treatment of BAY 11-7082 increased SK2 protein levels (Fig. 4, *A* and *B*), indicating that the regulation of SK2 expression by NF-κB occurred in the posttranslational levels. Furthermore, using siRNA technology, silencing of p65 caused a significant downregulation of Atrogin-1 mRNA and protein expression in HG cells (Fig. 4, *B* and *C*). To better understand the regulation of Atrogin-1 by the ROS–NF-κB axis, HL-1 cells with NG culture were treated with H_2_O_2_ (50 μM) after knocking down of the p65 gene. Western blotting analysis revealed that p65 knockdown reduced the effect of H_2_O_2_ on Atrogin-1 protein expression levels (Fig. 4*E*), by the evidence that a 50% decrease in p65 expression produced a 65% attenuation of H_2_O_2_ effects on Atrogin-1 expression (Fig. 4*E*), which suggests that NF-κB activation partially contributes to ROS-induced Atrogin-1 protein downregulation in HL-1 cells. Finally, the UCSC and JASPAR databases were used to predict p65 transcription factor binding sites on the Atrogin-1 promoter (TGGAGTTCCC) (Fig. S1). Chromatin immunoprecipitation (ChIP) analysis was used to assess the binding of the transcription factor p65 to the promoter sequence of *FBXO32* gene (encoding Atrogin-1) in HG-cultured cells. The results suggested that p65 binds to the promoter region of Atrogin-1 (Fig. 4, *F* and *G*). In summary, in diabetic atria NF-κB regulates Atrogin-1 expression is mediated through a direct control of *FBXO32* gene transcription.

### Regulation of atrial SK2 levels by Atrogin-1 is through the UPS in HG culture conditions

**Figure 5:**
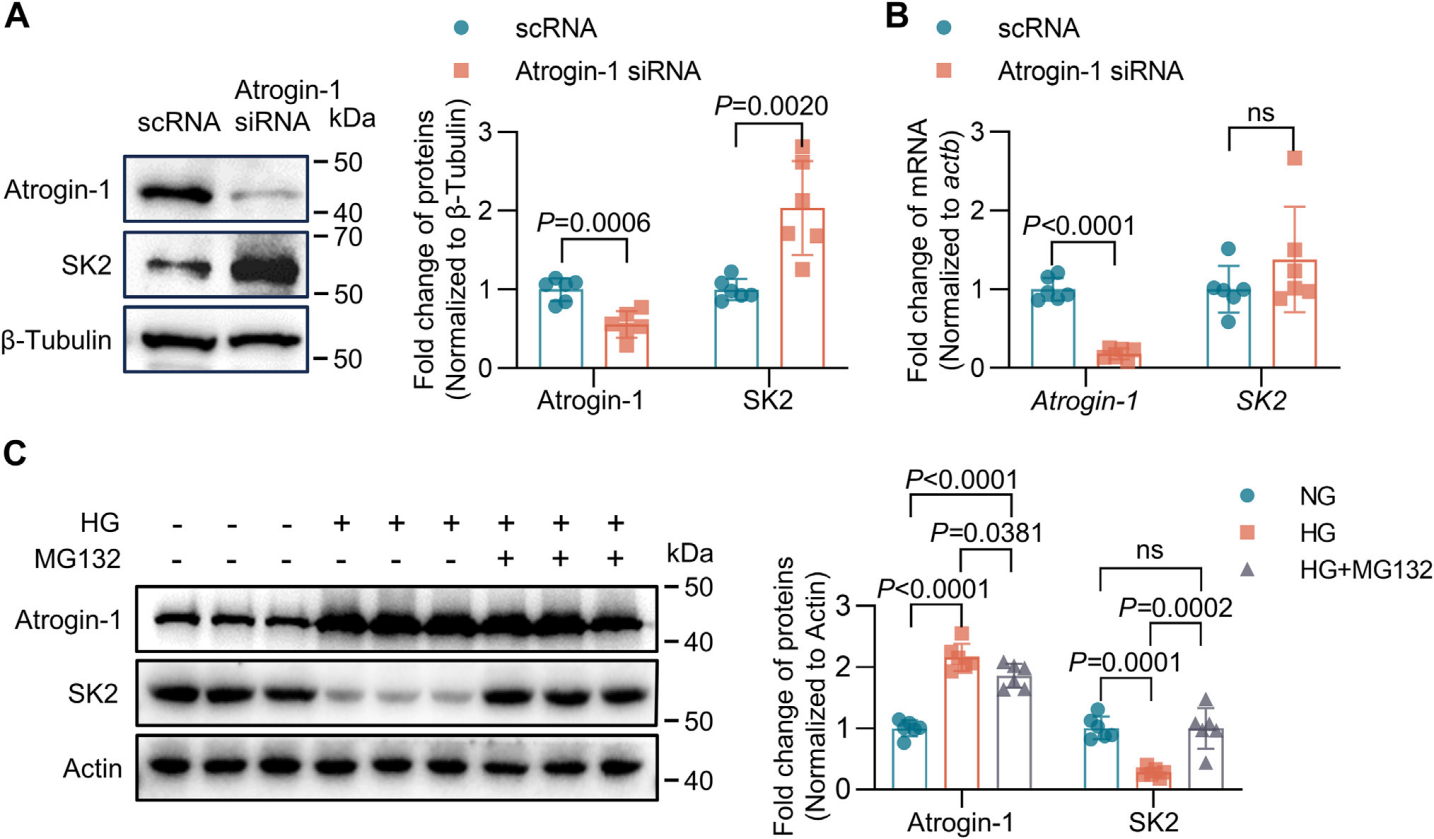
**Atrogin-1 and the UPS essentially regulate atrial SK2 levels in DM.***A* and *B*, application of Atrogin-1 siRNA for Atrogin-1 knockdown. *A*, Western blotting analysis to assess Atrogin-1 and SK2 protein expression (n = 6). *B*, RT-PCR analysis of Atrogin-1 and SK2 mRNA expression (n = 6). *C*, HL-1 cells were treated with MG132 (10 μM) for 24 h, and Western blotting analysis was performed to assess Atrogin-1 and SK2 protein expression (n = 6). Data are expressed as the mean ± SD and were assessed by unpaired, two-tailed Student’s *t* test, followed by Bonferroni correction (GraphPad Prism software). DM, diabetes mellitus; SK, small-conductance Ca^2+^-activated K^+^; UPS, ubiquitin‒proteasome system.

Previous studies have identified that downregulated SK2 channel protein levels in the atria of DM mice is associated with increased Atrogin-1 levels (6). To determine whether SK2 degradation is mediated through UPS, the Atrogin-1 expression and UPS function were suppressed using siRNA and MG132 (a cell-permeable proteasome inhibitor) in HL-1 cells. Silencing of the *FBXO32* gene resulted in an increase in SK2 protein expression, but not in its mRNA expression in HL-1 cells (Fig. 5, *A* and *B*). There was a remarkable increase in Atrogin-1 expression accompanied by a significant decrease in SK2 protein levels in HG-cultured HL-1 cells, compared to those in cells with NG culture. However, a 24-h incubation with 10 μM MG132 suppressed Atrogin-1 expression and restored SK2 protein expression against HG culture to the levels of the cells with NG culture (Fig. 5*C*).

### Curcumin reverses the effect of HG on Atrogin-1 expression

**Figure 6:**
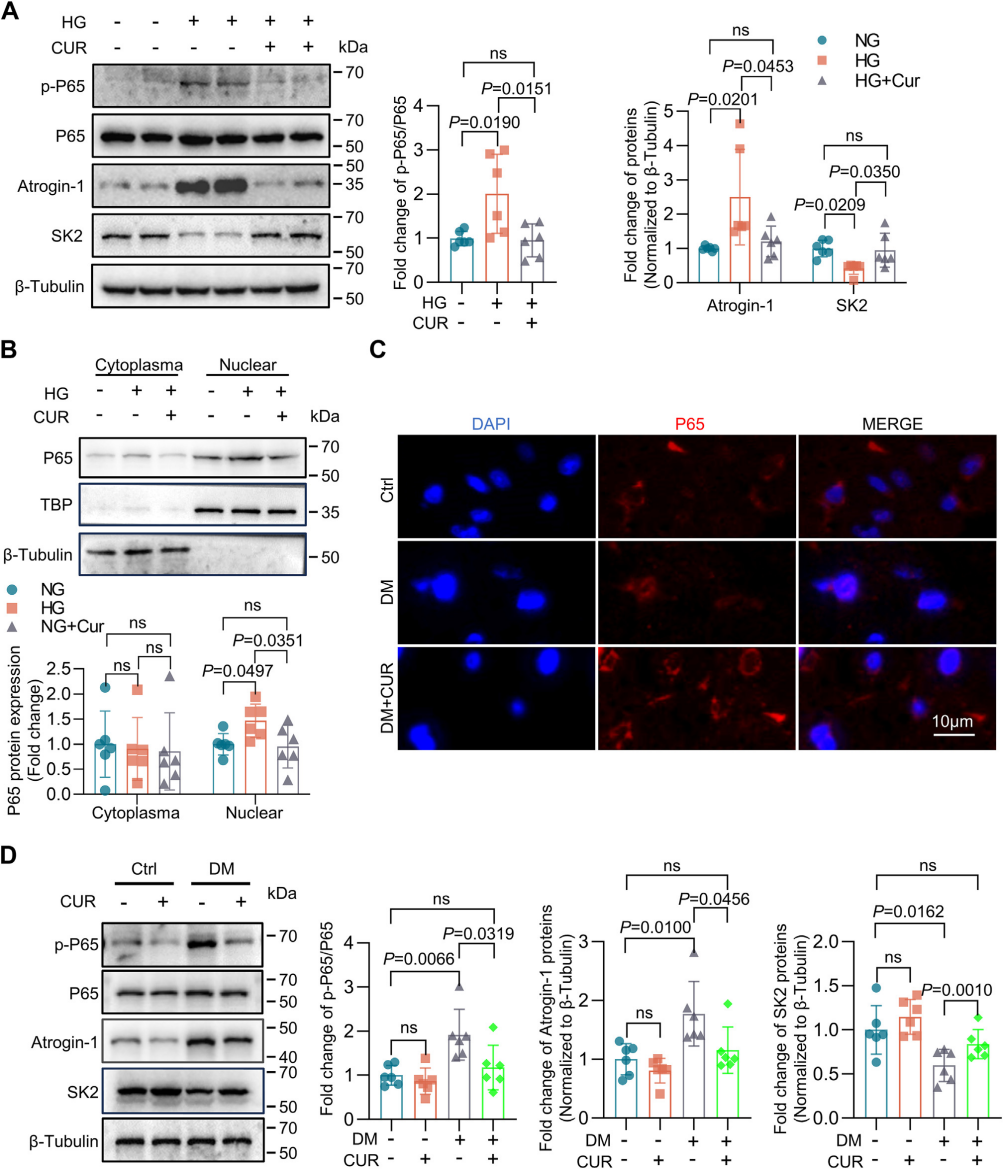
**Curcumin reverses the effect of HG on Atrogin-1.***A* and *B*, HG-cultured HL-1 cells were treated for 24 h with curcumin (20 μM); *A*, Western blotting analysis of p-p65 (Ser536), p65, Atrogin-1, and SK2 protein expression (n = 6). *B*, Western blotting analysis to assess p65 protein nuclear translocation (n = 6). *C* and *D*, for *in vivo* experiments with C57BL/6J mice, 200 mg/kg curcumin, o.p., was administered to STZ-induced DM mice once daily for 4 weeks. *C*, immunofluorescence analysis of p65 protein nuclear translocation (n = 6). *D*, Western blotting analysis of p-p65 (Ser536), p65, Atrogin-1, and SK2 protein expression (n = 6). Data are expressed as the mean ± SD. Data are expressed as the mean ± SD and were assessed by unpaired, two-tailed Student’s *t* test or one-way ANOVA, followed by Bonferroni correction (GraphPad Prism software). DM, diabetes mellitus; HG, high glucose; SK, small-conductance Ca^2+^-activated K^+^; STZ, streptozotocin.

It has been reported that curcumin inhibits the inappropriate activation of NF-κB (14, 15, 16) To determine the effect of curcumin on Atrogin-1 and SK2 expression, HL-1 cells propagated in HG were treated with curcumin (20 μM) for 24 h. This treatment led to the inhibition of p65 protein phosphorylation and its nuclear translocation, a key step of NF-κB signaling pathway activation. Additionally, it resulted in the downregulation of Atrogin-1 protein levels and the recovery of SK2 protein expression in HG culture conditions (Fig. 6, *A*–*C*). The effect of curcumin was also examined in DM mice 4 weeks after oral administration of curcumin (200 mg/kg, gavage once daily), showing a remarkable attenuation of p65 and Atrogin-1 protein expression, accompanied by a significant increase of SK2 expression in the mouse atrial myocytes (Fig. 6*D*). In summary, curcumin restores SK2 protein levels in the atria of DM mice, as results of inhibiting NF-κB–mediated *FBXO32* transcription and Atrogin-1–dependent SK2 protein degradation.

## Discussion

In this study, we have several important findings regarding the regulation of SK2 expression by ROS and Atrogin-1 in the context of diabetes. First, ROS stimulates the Atrogin-1–induced SK2 degradation through NF-κB signaling pathway in the atria of STZ-induced diabetic mice. Second, NF-κB/p65 upregulates Atrogin-1 expression through a direct binding to the *FBXO32* promoter and facilitates its transcription. Third, curcumin alleviates the effect of NF-κB signaling on Atrogin-1 expression by inhibiting the NF-κB/p65 phosphorylation and nuclear translocation in atrial cardiomyocytes.

SK2 channels are ubiquitously expression in many tissue beds, including those of nervous, vascular endothelia, skeletal muscles smooth muscles, and myocardia, especially in atria (17). Several studies have indicated a significant association between SK2 channels and AF (18, 19, 20). Although the precise role of SK channels in cardiac electrophysiology is not yet fully understood, there is evidence to suggest that they are proarrhythmogenic mediators for atrial electrical remodeling (18, 21). However, the role of SK channels on the development and maintenance of AF are controversial and both reduced and enhanced SK2 channel activity may predispose the atria to AF (22, 23, 24). Previous studies have suggested that inhibition of SK2 channels has antiarrhythmic effects in some animal models (22, 25). For example, inhibition of SK2 by NS8593 (a selective SK2 inhibitor) reduces the duration of burst pacing-induced AF in perfused atria of rats, guinea pigs, rabbits, and horses (25, 26). Additionally, short-term atrial burst pacing in rabbits increases SK2 levels at the plasma membrane, causing SK2 upregulation and consequent shortening of proarrhythmic action potential duration in rabbit pulmonary veins (27). Moreover, administration of NS8593 to rats with hypertension-induced atrial remodeling and dogs with atrial tachycardia-induced atrial remodeling decreases the duration of induced AF, validating the proarrhythmic role of enhanced SK2 in the remodeled atria of these animals (25). In clinical investigation, however, both upregulation and downregulation of SK channels are linked to AF in patients (22, 23, 24). We believe that the discrepancy varies from different disease status, AF models, species, and experimental conditions.

Multiple signaling pathways are involved in the regulation of Atrogin-1 expression. For example, the PI3K/AKT/FOXOs signaling pathway affects Atrogin-1 in muscle atrophy models through the FOXO family by recruiting Atrogin-1 promoter pairs and thus regulating its protein expression (28, 29). In the IL-6/JAK/STAT3 signaling pathway, STAT3 phosphorylation activates CCAAT/enhancer binding protein delta, increasing Atrogin-1 expression (30). It has been known that NF-κB signaling pathway may influence AF by regulating transcription and potentially directly affecting the sodium channel promoter, thereby increasing AF risk (31). We have previously reported that downregulation of SK2 prolongs the atrial action potential durations in DM mice, which is associated with an enhanced incidence of atrial arrhythmias (26). Moreover, decreased SK2 protein expression in DM atria was due to enhanced Atrogin-1 expression that induced SK2 degradation through the UPS pathway (6). In the present study, we provide strong evidence that Atrogin-1 expression is regulated by NF-κB/p65 and that increased ROS generation is the prime culprit of NF-κB/p65 activation in DM and HG culture conditions. We have identified for the first time that NF-κB/p65 directly binds to the Atrogin-1 promoter and enhances *FBXO32* transcription (Fig. 4), in turn affecting the SK2 channel expression in atrial myocardia. Pharmacological inhibition of ROS and NF-κB or genetic silencing of p65 decrease Atrogin-1 expression and increase SK2 protein levels in HG culture and DM. gene Hence, the results from our study demonstrate that the ROS/NF-κB signaling pathway plays a critical role in Atrogin-1–dependent SK2 protein degradation of atria.

Curcumin, a natural polyphenol extracted from turmeric root, has been found to exhibit antioxidant, antiapoptotic, anti-inflammatory, and anticancer effects (32). Curcumin has been shown to substantially reduce diabetic complications in experimental models, including alleviating cardiomyocyte apoptosis and improving heart function (33). Literatures have suggested that curcumin might protect the heart through various pathways (32, 34, 35), and it has been shown to be effective in AF treatment in a rat model (36); however, the underlying mechanism of its effect on AF is unclear. In this study, we confirm that oral administration of curcumin is capable to reduce Atrogin-1 protein expression induced by ROS-activated NF-κB signaling in atrial myocytes of STZ-induced DM mice. Whether curcumin treatment is beneficial to the patients with AF warrants further investigation.

In summary, in this study we have demonstrate that acceleration of SK2 protein degradation *via* UPS is mediated by NF-κB activation in response to increased ROS production in the atrial myocardia of DM. Specifically, NF-κB directly bound to the Atrogin-1 promoter and promoted *FBXO32* gene transcription, therefore accelerating Atrogin-1–dependnet SK2 protein degradation. Inhibition of NF-κB suppresses Atrogin-1 expression and protects SK2 protein levels in the DM. Hence, suppression of ROS/NF-κB/Atrogin-1 axis should be considered a therapeutic target for the treatment of atrial arrythmias in DM.

## Experimental procedures

### Diabetic mice

A diabetic mice model was established using C57BL/6J mice (6–8 weeks old) and the intraperitoneal administration of STZ (Sigma-Aldrich, S0130; 125 mg/kg body weight) (5). Control mice were given vehicle injections. After 4 weeks of injection, mice with blood glucose >16.9 mmol/L were deemed diabetic and utilized for subsequent experiments. All Experimental procedures were approved by the Animal Care and Use Committee of the Air Force Medical University (KY20193236). After 2 weeks of adaptation, the mice were randomly divided into a control (Ctrl) group and model (DM) group (untreated and treated, respectively). Half of these mice from each group were separated, and half were selected for the experiment. The mice in the treatment group received curcumin (MCE, HY-N0005, 200 mg/kg/d, o.p.), and the mice in the nontreatment group received an equal volume of solvent by gavage for 4 weeks. After 4 weeks, all mice in the Ctrl and DM groups were treated. Mice in the treatment group were administered DPI (MCE, HY-100965; 0.1 mg/kg/d) i.p., and mice in the nontreatment group were administered an equal volume of solvent for 2 weeks.

### Cell culture

A cardiac muscle cell lineage (HL-1 cells, InCellGene) was propagated in Dulbecco's Modified Eagle Medium (DMEM, HyClone) supplemented with 2 mM L-glutamine, 100 μg/ml streptomycin-penicillin, 0.1 mM norepinephrine, and 10% fetal bovine serum (InCellGene, IC-1905) in a humidified atmosphere with 5% CO_2_ at 37 °C. The media was replaced or replenished daily and upon confluency, the cells were enzymatically harvested and reseeded at a 1:4 dilution. HL-1 cells were propagated in DMEM containing either NG (5 mM) or HG (25 mM) for 2 weeks before the experiments. To further test our hypotheses, we applied H_2_O_2_ (50 μM) to treat HL-1 cells in NG culture for 24 h; DPI (10 μM) to treat HL-1 cells in HG culture for 24 h; BAY 11-7082 (5 μM) to treat HL-1 cells in HG culture for 24 h; curcumin (20 μM) to treat HL-1 cells in HG culture for 24 h; and MG132 (10 μM) to treat HL-1 cells for 24 h (Table S1).

### Real-time quantitative PCR

Total RNA from HL-1 cells and atrial tissues was acquired using TRIzol reagent following the manufacturer’s instructions (TIANGEN, DP421). Complementary DNA was synthesized with TIANScript complementary DNA kits (TIANGEN, KR104) and real-time PCR with a Cycler Sequence Detection System (Bio-Rad, CFX96). The PCR cycle was as follows: 40 cycles at 95 °C for 10 s, 55 °C for 20 s, and 72 °C for 20 s. The relative mRNA levels were assessed using the 2^−ΔΔCt^ method and normalized to actb. The primer sequences are available in Table S2.

### Western blotting

Total protein was acquired from HL-1 cells and atrial tissues and prepared using the nuclear and cytoplasmic extraction kit (Beyotime, P0027). Proteins were separated by gel electrophoresis and transferred to a polyvinylidene fluoride membrane. The membrane was blocked for 1 h in 5% milk, incubated overnight in primary antibodies (4 °C), rinsed with TBST (RSKBIO; RS5106), and incubated for 1 h with HRP-conjugated secondary antibodies (InCellGene; SA-10011, 1:5000 dilution). Finally, images were acquired using the Molecular Imager ChemiDoc XRS + System (Bio-Rad). Band densitometry was quantified by Quantity One software (Bio-Rad; https://www.bio-rad.com/zh-cn/product/quantity-one-1-d-analysis-software?ID=1de9eb3a-1eb5-4edb-82d2-68b91bf360fb). The details of primary antibody information can be found in Table S3.

### siRNA transfection

HL-1 cells were transfected with p65 (RelA) and Atrogin-1 (FXBO32) siRNA and negative control siRNA *via* Lipofectamine 2000. After plating, at 60∼80% confluency, cells were serum-starved in serum-free DMEM for 2 h. Then, siRNA (100 nM) was transfected (50 μM) into cells in serum-free medium. After 6 h of incubation, the culture medium was discarded and replaced with complete media containing 10% serum, and the cells were incubated for 48 h before subsequent experiments. Table S4 provides the details of the sequences of the primers used.

### Chromatin immunoprecipitation

As per guided by the Cell Signaling Technology kit (catalog number 9003), HL-1 cells (5 × 10^7^) were propagated in HG conditions for 14 days and then cross-linked with 1% formaldehyde in DMEM at room temperature. After 10 min, 125 mM glycine was added to terminate cross-linking, and then, 2 μl of micrococcal nuclease was added for 20 min at 37 °C. A sonicator (Diagenode) was set to 30 W to sonicate the cross-linked samples (ten times, 6 s each). The chromatin length was analyzed by agarose gel electrophoresis, and the segments ranged between 150 and 900 bp. Cross-linked chromatin fragments (10 μg of DNA) were immunoprecipitated with ChIP-grade p65 (#8242), IgG (#2729), and H3 (#4620) antibodies overnight at 4 °C. ChIP-grade protein G magnetic beads were used to recover the complexes. DNA was purified. The Atrogin-1 promoter sequence was acquired from the UCSC website, and based on this sequence, ten primer sequences were designed (Table S5). After PCR amplification, the DNA products were analyzed in a 1.5% agarose gel. Finally, CT values of each product were acquired *via* qPCR, and the enrichment relative to the input was calculated:

$$\text{Enrichment}\;\text{relative}\;\text{to}\;\text{input}\;\left(\%\right)=\frac{E{\left(\text{target}\right)}^{\Delta C_{q}\left(Input-IP\right)}}{E{\left(\text{positive}\right)}^{\Delta C_{q}\left(Input-IP\right)}}X\;100\%$$

Then, the enrichment percentages were used to determine the occupancy relative to the control. These relative values were then used for a *t* test. p65 and Atrogin-1 interactions were then assessed.

### Immunofluorescence staining

Briefly, waxed samples were sliced (5 μm), placed on glass slides, stained, deparaffinized, subjected to hot citric acid buffer for antigen retrieval, cooled, permeabilized using 0.2% Triton X-100 for 15 min, blocked for 2 h with 1% BSA (G-Clone, PN4810) in PBS, incubated with primary antibodies overnight (4 °C), and then incubated with Alexa Fluor 594–linked secondary antibodies. The nuclei of cells and embedded tissue were stained with 4′,6-diamidino-2-phenylindole (Vector Laboratories, H-1200). An Olympus BX51 Fluorescence Microscope and an Olympus DP72 camera were used to acquire all immunostaining micrographs.

### Measurement of intracellular ROS generation

Measurement of intracellular (HL-1) ROS levels: as instructed by the ROS Assay Kit (Beyotime, S0033S), briefly, after 14 days of HL-1 cell propagation in NG and HG culture conditions, the cells were seeded in 12-well culture dishes. After 24 h of DPI (MCE, HY-100965) treatment in HG culture, dichlorofluorescein diacetate (10 μmol) in serum-free culture medium was added to the cells in the dark for 1 h at 37 °C. Then, the cells were rinsed three times and photographed using a laser confocal microscope. The ROS fluorescence intensity was calculated using ImageJ software (https://imagej.net/downloads).

Measurement of ROS levels in atria: after treatment, atrial tissues from the control, diabetic, and drug intervention groups were taken for frozen sectioning. Dihydroethidium (2 μM) was applied topically to each tissue section for 30 min in the dark at 37 °C. An orthofluorescence microscope was used to collect images (Nikon Eclipse C1, Nikon) after the addition of an antifluorescence quenching sealer (CY3 excitation wavelength, 510–560 nm; emission wavelength, 590 nm).

### Statistical analysis

Data were assessed by the unpaired, two-tailed Student’s *t* test (for two groups) or ANOVA (for ≥3 groups). Bonferroni correction was carried out with GraphPad Prism software (GraphPad, 8; https://www.graphpad-prism.cn/), and the data are presented as the mean ± SD.

## Data availability

The data that support the findings of this study are available on request from the corresponding author, Fu Yi, upon reasonable request.

## Supporting information

This article contains supporting information.

## Conflict of interest

The authors declare that they have no no conflicts of interest with the contents of this article.
