# Supplementary material for: ROS in diabetic atria regulate SK2 degradation by Atrogin-1 through the NF-κB signaling pathway
Source: J Biol Chem. 2024 Feb 8;300(3):105735. doi: 10.1016/j.jbc.2024.105735 (PMC10938124; doi:10.1016/j.jbc.2024.105735)
Supplement: Supplementary Table [file mmc1.pdf]

**OS in diabetic atria regulates SK2 degradation by Atrogin-1 through the NFκB signaling pathway**

Jian Xu[1,2], Dong Zhang[1], Yibo Ma[1], Hui Du[1], Yi Wang[1], Wenping Luo[3], Ruxing Wang[4], Fu Yi[1]*.

[1]Department of Cardiovascular Diseases, Xijing Hospital, Fourth Military Medical University, Xi’an 710032, China;

[2]Department of Cardiology, The Sixth Medical Centre, Chinese PLA General Hospital, Beijing 100048, China

[3]Institute of Cardiovascular and vascular Disease, Shaanxi University of Traditional Chinese Medicine, Xianyang 712000, China;

[4]Department of Cardiology, Wuxi People's Hospital Affiliated to Nanjing Medical University, Wuxi, Jiangsu 214023, China.

***Correspondence to：** yi12fu56@126.com

**This PDF file includes:**

Supplementary tables and figures

**Supplementary tables**

**Supporting Table for Figure 1 E1**

| group | actb | NFKB1 | P65 | Atrogin-1 | SK2 |
|---|---|---|---|---|---|
| Ctrl | 19.25 | 28.50 | 27.59 | 27.28 | 27.04 |
| Ctrl | 19.52 | 28.31 | 27.65 | 26.85 | 26.85 |
| Ctrl | 20.20 | 29.17 | 28.62 | 28.08 | 27.49 |
| DM | 20.57 | 29.48 | 28.93 | 27.96 | 28.76 |
| DM | 20.26 | 29.21 | 28.33 | 27.03 | 28.47 |
| DM | 25.44 | 31.24 | 32.27 | 31.86 | 33.39 |

**Supporting Table for Figure 1 E2**

| group | actb | NFKB1 | P65 | Atrogin-1 | SK2 |
|---|---|---|---|---|---|
| Ctrl1 | 20.22 | 29.90 | 28.34 | 27.39 | 28.22 |
| Ctrl1 | 20.32 | 29.92 | 28.11 | 27.37 | 28.03 |
| Ctrl1 | 20.25 | 29.85 | 28.27 | 27.52 | 28.20 |
| Ctrl2 | 20.78 | 29.64 | 28.31 | 27.37 | 28.69 |
| Ctrl2 | 20.48 | 29.54 | 28.23 | 27.34 | 28.61 |
| Ctrl2 | 20.56 | 29.76 | 28.29 | 27.43 | 28.56 |
| Ctrl3 | 21.12 | 30.51 | 29.12 | 28.43 | 29.51 |
| Ctrl3 | 20.91 | 30.34 | 28.70 | 28.57 | 29.35 |
| Ctrl3 | 21.01 | 30.48 | 29.01 | 28.72 | 29.25 |
| DM1 | 23.59 | 30.67 | 29.68 | 27.95 | 30.56 |
| DM1 | 23.48 | 30.20 | 29.84 | 28.03 | 30.73 |
| DM1 | 23.42 | 29.64 | 29.34 | 28.02 | 30.54 |
| DM2 | 21.96 | 29.36 | 28.33 | 26.62 | 29.02 |
| DM2 | 21.96 | 29.55 | 28.42 | 26.71 | 28.76 |
| DM2 | 22.07 | 29.51 | 28.23 | 26.87 | 28.79 |
| DM3 | 23.83 | 31.04 | 30.20 | 29.05 | 32.27 |
| DM3 | 23.80 | 31.04 | 29.99 | 29.47 | 31.95 |
| DM3 | 23.92 | 31.17 | 30.03 | 29.06 | 31.57 |

**Table S1.** Supporting Table for Figure 1 E. RT-PCR analysis of Ctrl vs. DM groups for NFκB1, P65 (RELA), Atrogin-1 (FBXO32), SK2 expression

**Supporting Table for Figure 2 B1**

| group | actb | Atrogin-1 | SK2 | P65 |
|---|---|---|---|---|
| NG | 17.28 | 25.40 | 27.30 | 29.11 |
| NG | 16.95 | 25.35 | 27.32 | 29.19 |
| NG | 16.91 | 25.19 | 26.89 | 29.05 |
| HG | 17.49 | 24.76 | 27.17 | 28.38 |
| HG | 17.20 | 24.58 | 27.18 | 28.36 |
| HG | 17.12 | 24.52 | 27.05 | 28.34 |
| NG+$H_2O_2$ | 16.98 | 24.40 | 27.30 | 27.24 |
| NG+$H_2O_2$ | 16.95 | 24.35 | 27.32 | 27.31 |
| NG+$H_2O_2$ | 16.91 | 24.19 | 26.89 | 27.59 |

**Supporting Table for Figure 2 B2**

| group | actb | Atrogin-1 | SK2 | P65 |
|---|---|---|---|---|
| NG | 16.75 | 24.49 | 26.67 | 25.77 |
| NG | 16.75 | 24.16 | 26.71 | 25.62 |
| NG | 16.80 | 24.43 | 26.71 | 25.59 |
| HG | 17.49 | 24.70 | 27.15 | 25.81 |
| HG | 17.17 | 24.28 | 27.06 | 25.58 |
| HG | 17.07 | 23.98 | 27.06 | 25.22 |
| NG+$H_2O_2$ | 17.82 | 23.93 | 27.45 | 25.54 |
| NG+$H_2O_2$ | 17.35 | 23.53 | 27.28 | 25.54 |
| NG+$H_2O_2$ | 17.41 | 23.53 | 27.39 | 25.55 |

**Table S2.** Supporting Table for Figure 2 B. HL-1 cells were propagated in HG media for 14 days and were stimulated with 20 μM $H_2O_2$ for 24 h before RT-PCR analysis. P65 (*RELA*), Atrogin-1 (*FBXO32*), SK2 expression by RT-PCR after 24 h of H2O2 stimulation

**Supporting Table for Figure 3 B1**

| group | actb | p65 |
|---|---|---|
| NG | 17.02 | 27.69 |
| NG | 17.03 | 27.83 |
| NG | 16.74 | 28.25 |
| HG | 18.34 | 26.88 |
| HG | 18.13 | 27.05 |
| HG | 18.27 | 27.13 |
| DPI | 16.67 | 27.58 |
| DPI | 17.16 | 27.56 |
| DPI | 16.66 | 27.65 |
| CUR | 20.35 | 29.57 |
| CUR | 20.27 | 29.50 |
| CUR | 17.95 | 29.50 |

**Supporting Table for Figure 3 B2**

| group | actb | Atroin-1 |
|---|---|---|
| NG | 18.62 | 29.07 |
| NG | 18.2641 | 27.0214 |
| NG | 18.3494 | 27.886 |
| HG | 17.47 | 25.14 |
| HG | 17.29 | 25.94 |
| HG | 18.36 | 26.20 |
| DPI | 18.20 | 28.06 |
| DPI | 18.21 | 27.51 |
| DPI | 19.53 | 27.76 |
| CUR | 18.24 | 27.80 |
| CUR | 19.30 | 28.28 |
| CUR | 18.49 | 27.78 |

**Table S3.** Supporting Table for Figure 3 B. RT-PCR analysis of P65 (*RELA*), Atrogin-1 (*FBXO32*) mRNA expression

**Supporting Table for Figure 4 A1**

| group | actb | Atrogin-1 | P65 |
|---|---|---|---|
| HG | 17.83 | 28.03 | 26.47 |
| HG | 17.16 | 28.03 | 26.13 |
| HG | 17.28 | 27.81 | 26.07 |
| BAY | 17.51 | 28.30 | 26.25 |
| BAY | 18.30 | 28.10 | 26.12 |
| BAY | 18.19 | 28.06 | 26.08 |

**Supporting Table for Figure 4 A2**

| group | actb | Atrogin-1 | P65 |
|---|---|---|---|
| HG | 16.21 | 25.31 | 23.35 |
| HG | 16.69 | 25.33 | 23.73 |
| HG | 16.61 | 25.40 | 23.62 |
| BAY | 16.65 | 26.38 | 24.11 |
| BAY | 16.57 | 26.51 | 24.17 |
| BAY | 16.60 | 26.46 | 24.32 |

**Table S4.** Supporting Table for Figure 4 A. HG cultured HL-1 cells were given BAY11-7082 (5 μM) intervention for 12 h. RT-PCR analysis of P65 (*RELA*), Atrogin-1 (*FBXO32*), SK2 mRNA expression

**Supporting Table for Figure 4 G**

| group | 2%input | IgG | P65 |
|---|---|---|---|
| 1 | 23.93 | 25.37 | 23.57 |
| 2 | 23.79 | 25.30 | 23.56 |
| 3 | 23.65 | 25.30 | 23.92 |
| 4 | 23.70 | 25.41 | 23.69 |
| 5 | 23.78 | 25.42 | 23.67 |
| 6 | 23.68 | 25.50 | 23.68 |

**Table S5.** Supporting Table for Figure 4 G. CHIP-qPCR analysis of Atrogin-1 (*FBXO32*) promoter sequence enriched ploidy

**Supporting Table for Figure 5 B1**

| group | actb | Atrogin-1 | SK2 |
|---|---|---|---|
| scRNA | 18.43 | 29.12 | 33.62 |
| scRNA | 18.54 | 29.01 | 33.01 |
| scRNA | 18.51 | 28.63 | 32.25 |
| Atrogin-1 siRNA | 18.32 | 32.41 | 32.10 |
| Atrogin-2 siRNA | 18.33 | 31.63 | 32.39 |
| Atrogin-3 siRNA | 18.33 | 31.57 | 31.27 |

**Supporting Table for Figure 5 B2**

| group | actb | Atrogin-1 | SK2 |
|---|---|---|---|
| scRNA | 17.87 | 28.59 | 25.81 |
| scRNA | 18.06 | 28.61 | 26.20 |
| scRNA | 17.92 | 28.57 | 26.07 |
| Atrogin-1 siRNA | 17.63 | 28.24 | 27.74 |
| Atrogin-2 siRNA | 17.61 | 28.18 | 27.73 |
| Atrogin-3 siRNA | 17.61 | 28.38 | 27.58 |

**Table S6.** Supporting Table for Figure 5 B. RT-PCR analysis of Atrogin-1 and SK2 mRNA expression

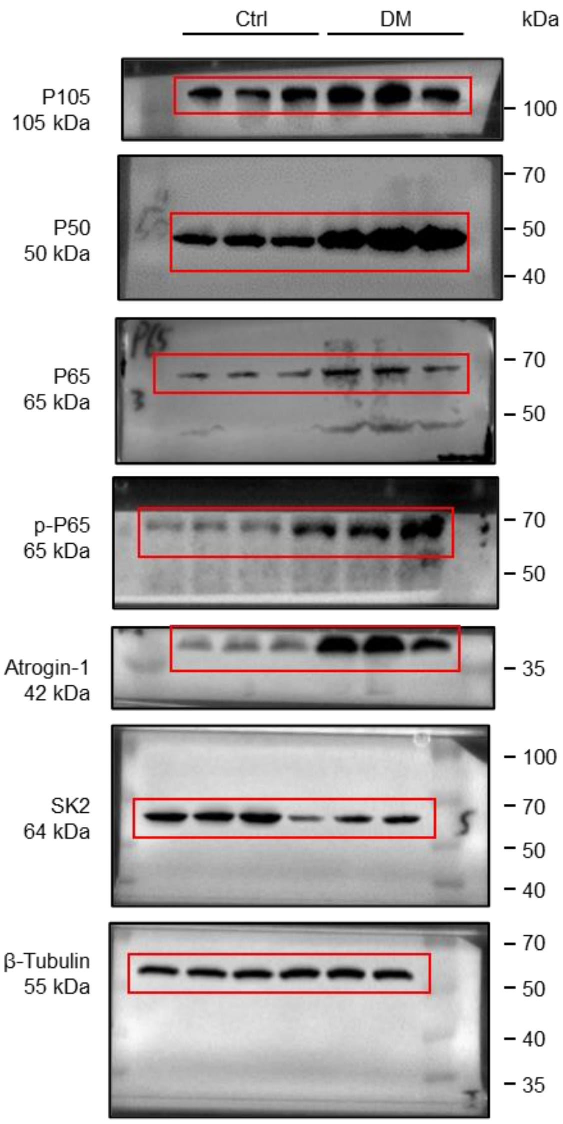


**Fig. S1.** Supporting Figure 1 D. Western blot analysis of Ctrl vs. DM groups for P105, P50, P65, p-P65, Atrogin-1, SK2 protein expression.

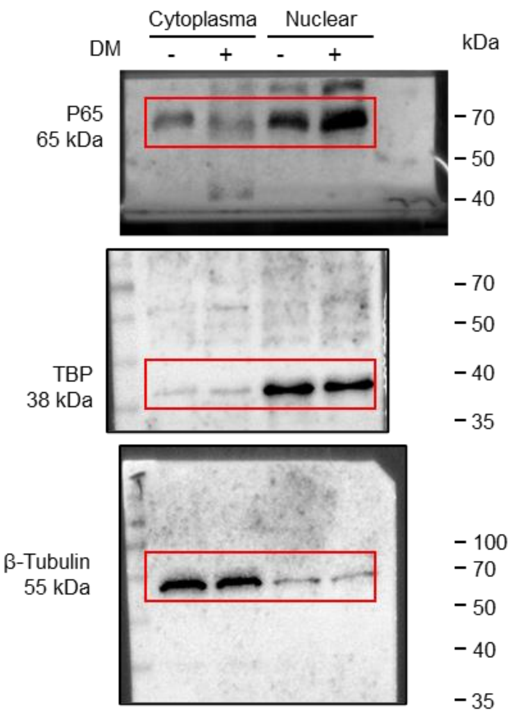


**Fig. S2.** Supporting Figure 1 F. Western blot analysis of P65 protein nucleoplasmic translocation in the Ctrl vs. DM groups

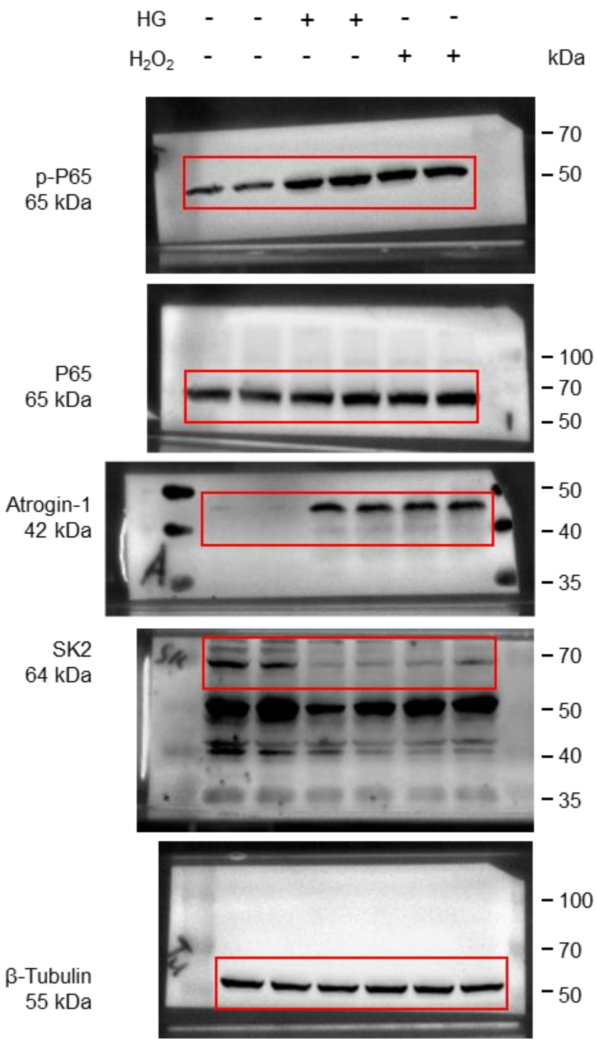


**Fig. S3**. Supporting Figure 2 C. Western blot analysis of pP65 (Ser536), P65, Atrogin-1 with SK2 protein expression

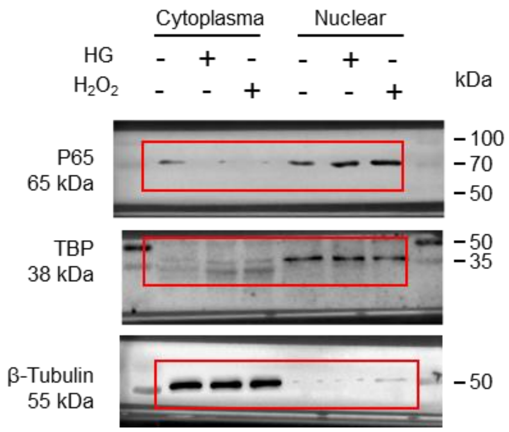


**Fig. S4.** Supporting Figure 2 D. Western blot analysis of P65 nuclear translocation

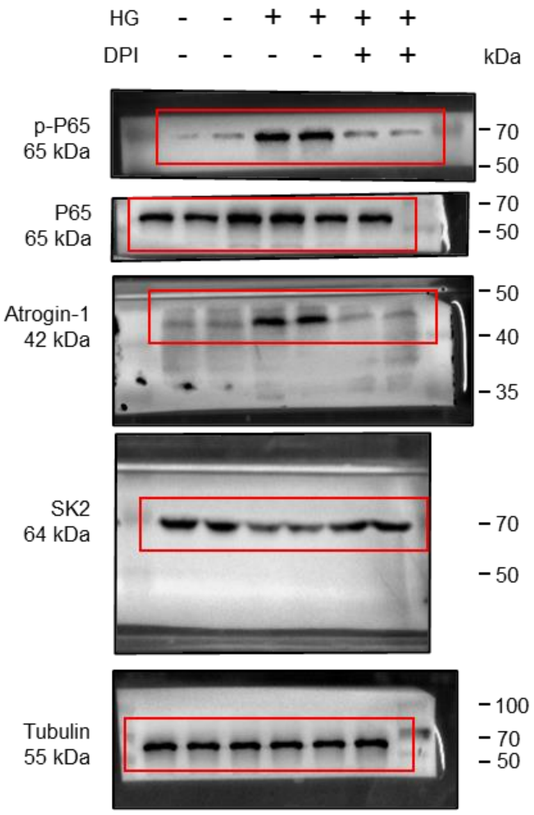


**Fig. S5.** Supporting Figure 3 C. Western blot analysis of pP65 (Ser536), P65, Atrogin-1 with SK2 protein expression

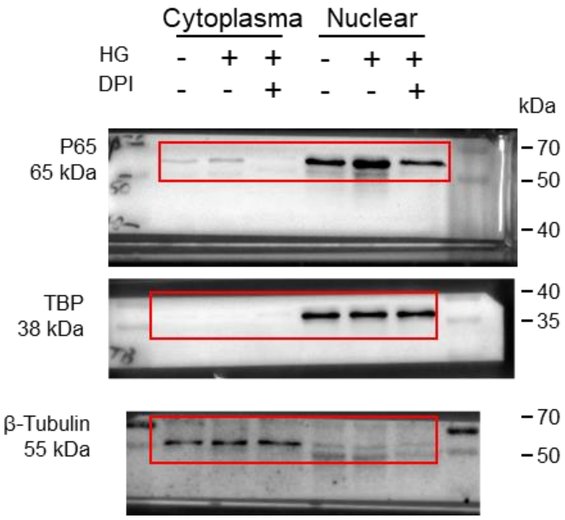


**Fig. S6.** Supporting Figure 3 D. Western blot analysis of intra-nuclear P65 expression

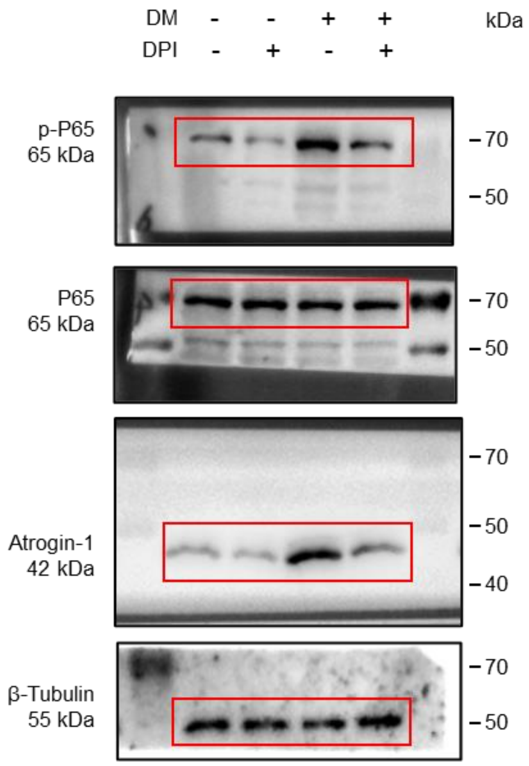


**Fig. S7.** Supporting Figure 3 G. Western blot analysis of DPI on pP65 (Ser536), P65, Atrogin-1 protein expression in mouse atrial muscle

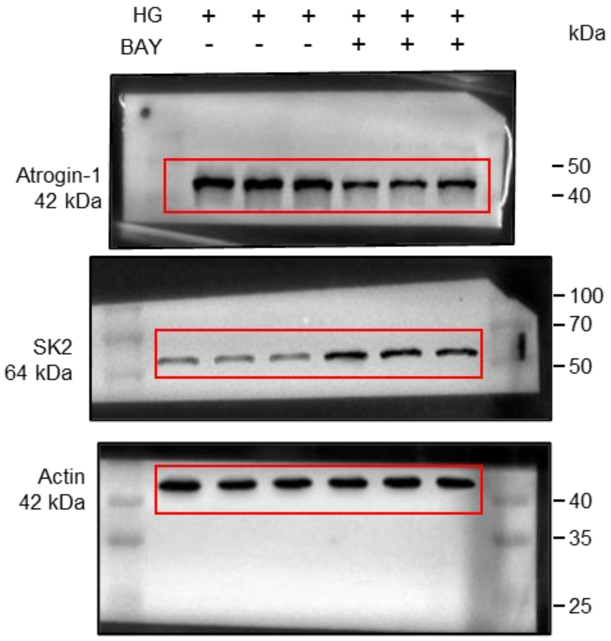


**Fig. S8.** Supporting Figure 4 B. Western blot analysis of BAY11-7082 on Atrogin in HG cultured HL-1 cells -1, SK2 protein expression

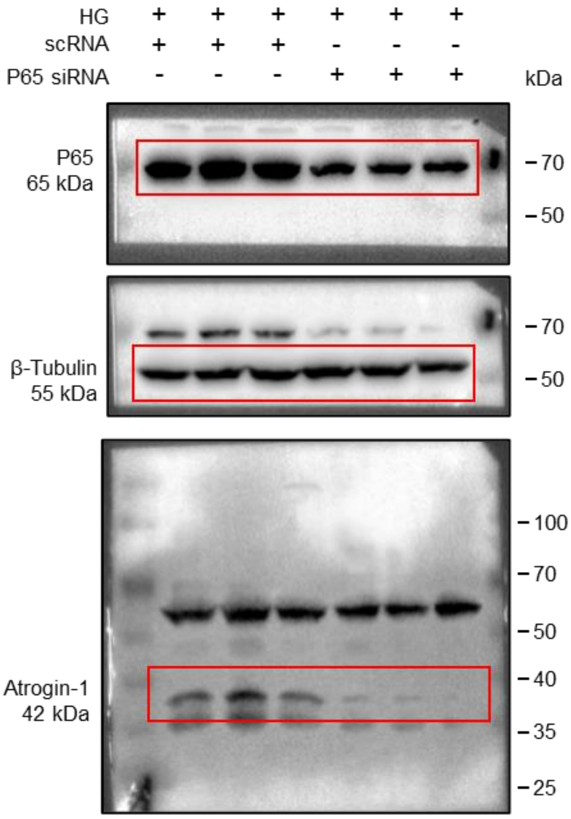


**Fig. S9.** Supporting Figure 4 D. Western blot analysis of P65, Atrogin-1 after P65 gene knockdown protein expression

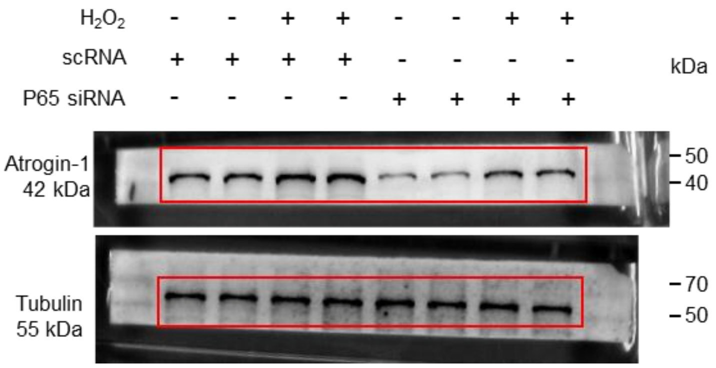


**Fig. S10.** Supporting Figure 4 E. NG cultured HL-1 cells were treated with P65 siRNA for 48 h and then with $H_2O_2$ (50 μM) for 24 h. Western blot analysis of Atrogin-1 protein expression

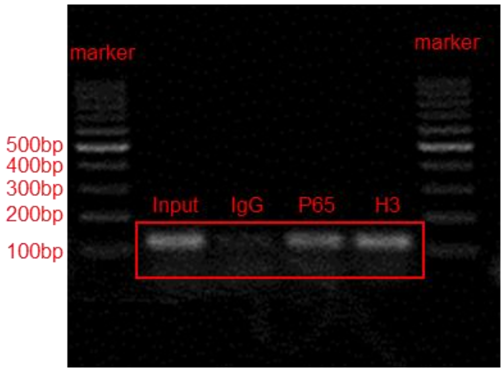


**Fig. S11.** Supporting Figure 4 F. PCR amplification followed by agarose gel electrophoresis analysis of Atrogin-1 (*FBXO32*) promoter sequence enriched fragments

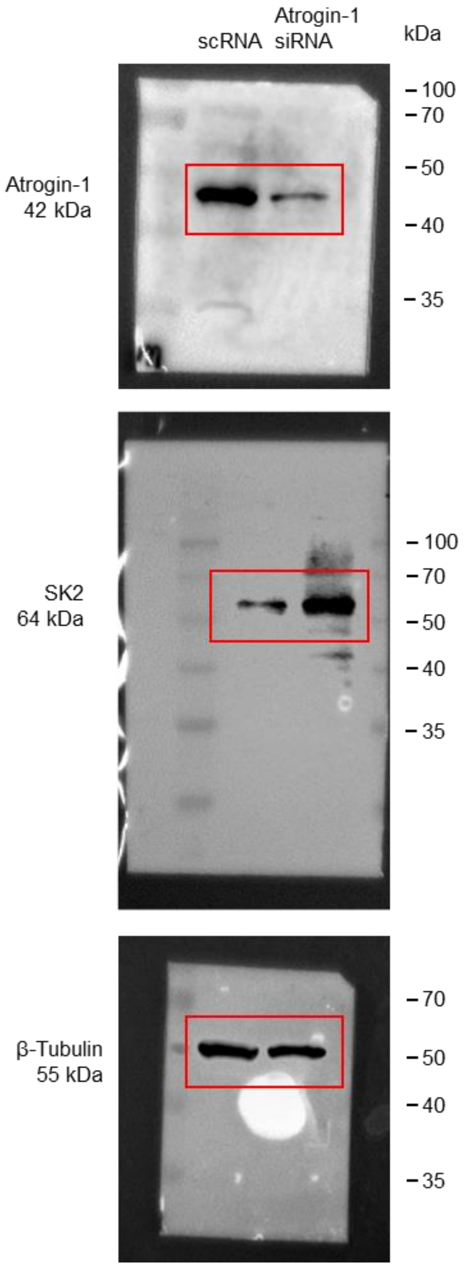


**Fig. S12.** Supporting Figure 5 A. Western blotting for assessing Atrogin-1 and SK2 protein expression

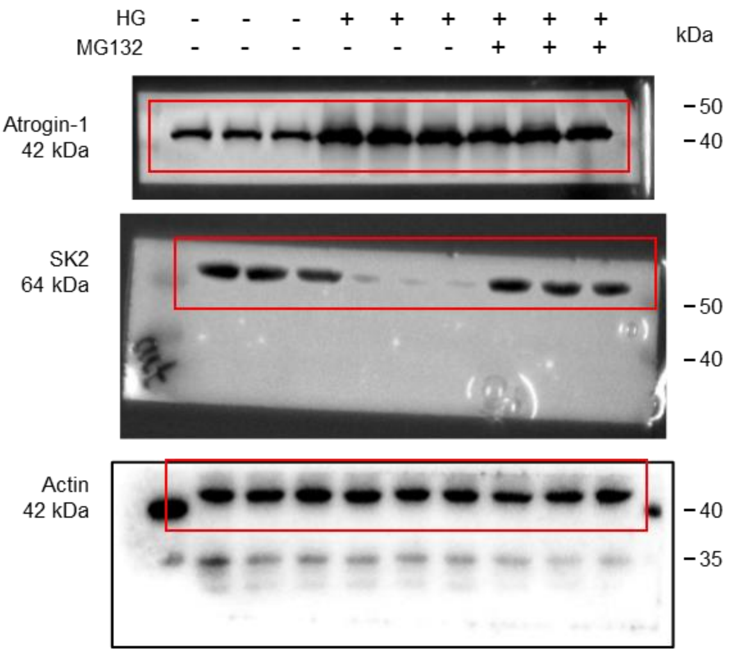


**Fig. S13.** Supporting Figure 5 C. HL-1 cells were treated with MG132 (10 μM) for 24h, Western blotting for elucidation of Atrogin-1 and SK2 protein expression

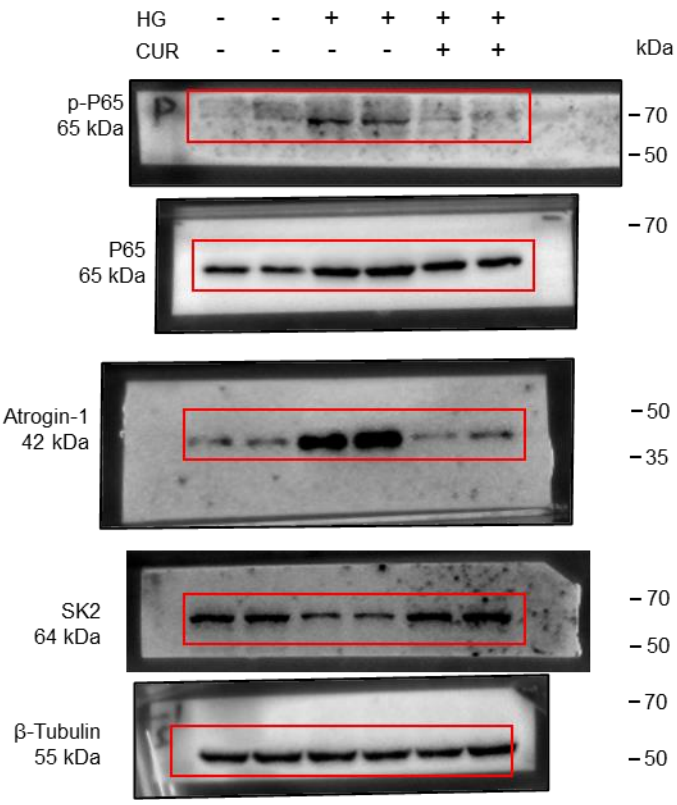


**Fig. S14.** Supporting Figure 6 A. Western blotting evaluation of p-P65 (Ser536), P65, Atrogin-1, and SK2 protein expression

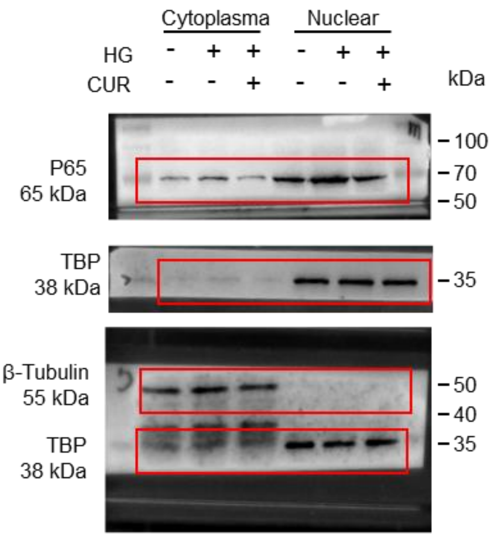


**Fig. S15.** Supporting Figure 6 B. Western blot for identifying P65 protein nuclear translocation (n = 6); C-D. For C57 mice in vivo experiment, 200 mg/Kg Curcumin, o.p. was given to STZ-induced DM mice once daily for 4 weeks

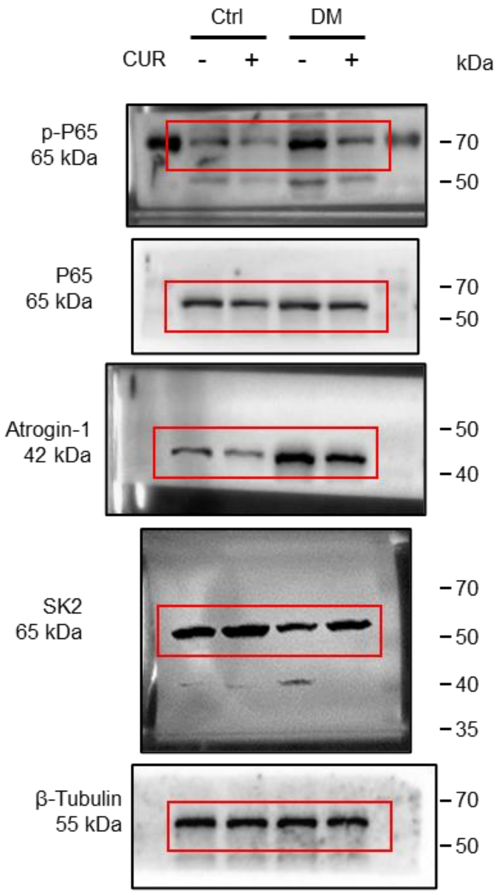


**Fig. S16.** Supporting Figure 6 D. Western blot analysis of p-P65 (Ser536), P65, Atrogin-1, SK2 protein expression
